# Examining the role of social factors in the utilization of preventive cardiovascular clinical services: An analysis using Andersen’s behavioral health model

**DOI:** 10.1371/journal.pone.0341074

**Published:** 2026-01-23

**Authors:** Sultan Saad Alamri, Abuobieda Khogali Abdalrouf, Fahad Mustafa Alnouri, Ammar Hamid Suliman

**Affiliations:** 1 Preventive Medicine Division, Department of Family and Community Medicine, Prince Sultan Military Medical City, Riyadh, Saudi Arabia; 2 Department of Family and Community Medicine, Prince Sultan Military Medical City, Riyadh, Saudi Arabia; 3 Cardiovascular Prevention Unit, Prince Sultan Cardiac Center, Riyadh, Saudi Arabia; National University, SUDAN

## Abstract

**Background:**

Cardiovascular disease (CVD) remains the leading cause of mortality in Saudi Arabia, mainly influenced by multiple lifestyle risk factors. Addressing this challenge requires a comprehensive evaluation of preventive services, particularly Preventive Cardiovascular Clinical Services (PCCS), which are essential for early detection and management. Hence, studying the utilization of PCCS is crucial to assess uptake and inform strategies to combat CVD burden.

**Methods:**

A survey-based cross-sectional study was conducted at the largest primary healthcare center under the Armed Forces Healthcare Services (AFHS) in Riyadh, Saudi Arabia. We surveyed 384 respondents to assess predisposing, enabling, and need factors that influence the utilization of nine PCCS using Andersen’s Behavioral Model of Health Services Use. Descriptive statistics and logistic regression models were employed to explore predictors of service utilization.

**Results:**

The study revealed a notably high prevalence of both positive family history (90%) and self-reported diagnosis (53%) of CVD among participants, highlighting significant disparities in the utilization of PCCS across different service types and participant characteristics. Predisposing factors, including sex, age, parenthood, and educational attainment, consistently influenced the utilization of various PCCS. Sex-specific disparities were observed, with female individuals engaging in more PCCS, while gaps persisted in services such as smoking-cessation counseling. Personal and organizational enabling factors, including vehicle ownership and proximity to the nearest primary healthcare center, were crucial in facilitating access to healthcare. Finally, need factors, such as family history of CVD, the presence of CVD risk factors, and perceived health status, strongly influenced individuals’ motivations for seeking PCCS.

**Conclusion:**

The complex intertwining of multiple personal, structural, and contextual factors further underscores the need to enhance PCCS user experience by designing, implementing, and monitoring targeted interventions, particularly for high-risk groups, based on their predisposing, enabling, and need profiles.

## Introduction

Cardiovascular disease (CVD) accounts for 32% of all global deaths and remains the leading cause of mortality worldwide [1]. In Saudi Arabia, CVD is responsible for 35.7% of all deaths [2], and accounts for 4,010 disability-adjusted life-years (DALYs) per 100,000 population [3], imposing a significant economic burden projected to reach 9.8 billion U.S. dollars in both direct and indirect health costs by 2035 [4]. Primary and secondary preventive measures effectively reduce CVD risk factors [5, 6], making them an essential component in combating the national burden of non-communicable diseases. In alignment, the Health Sector Transformation Program has launched several initiatives with a strong focus on preventive care [7]. Such initiatives extend beyond the public healthcare sector to actively engage the private one, allowing for a free-of-charge essential preventive care benefits package that includes obesity management using medical and surgical means [8]. A recent report by the National Heart Center (NHC) recommended establishing a comprehensive national CVD program encompassing risk screening, region-specific guidelines and monitoring tools, specialized training for healthcare providers, along with public awareness campaigns [9]. Notably, an economic model used in the same report suggested that implementing and monitoring such recommendations could prevent 112,748 deaths and 465,378 non-fatal CVD events within 5 years [9].

To assess the impact of many healthcare interventions, researchers often use utilization of healthcare services as a measure of effectiveness [10]. Healthcare utilization refers to the use of healthcare services by individuals for preventing and/or treating conditions, promoting wellness, or obtaining information about their health status or prognosis [11]. Healthcare utilization research seeks to understand the reasons of overusing, underusing, or misusing healthcare services. Beyond utilization metrics, one of the most widely used frameworks for studying healthcare service utilization is the Behavioral Model of Health Service Utilization (BMHSU) developed in 1968 by Roland M. Andersen [12]. This framework argues that individual and contextual factors influence individuals’ health behaviors when interacting with the healthcare system [13]. The model focuses on three core components; predisposing factors (demographics and social structures, e.g., age, sex, education, marital status), enabling factors (service-use enablers, e.g., available transportation, distance to healthcare centers), and need factors (factors that motivate service use, e.g., perceived need for physicians’ assessment) [14]. This framework helps explain factors that can facilitate or hinder access to healthcare [15]. Each element can be traced back to individual (e.g., genetics) or local contextual determinants (e.g., cultural norms) [13].

This research was conducted at the largest primary healthcare center (PHC) under Prince Sultan Military Medical City (PSMMC) [16]. PSMMC’s mission focuses on promoting healthy lifestyles and preventive medical services [17]. Its strategic goals include achieving a 90% score on the Preventive Services Index by 2024 through initiatives targeting major non-communicable diseases, such as screening clinics, rehabilitations centers, and updated clinical guidelines.

This study aims to identify factors influencing the utilization of Preventive Cardiovascular Clinical Services (PCCS) by employing Andersen’s behavioral model as a conceptual framework for analyzing healthcare utilization at the primary care level. The model facilitates the identification of service gaps and population characteristics to inform and implement targeted interventions.

### Literature survey

The BMHSU was developed to examine and predict individuals’ behaviors for utilizing healthcare services [18]. The model has been revised multiple times, with its latest version released in 2013 [19]. The three components of the model have been extensively studied in healthcare utilization research.

A study analyzing data from the National Health Interview Survey using Andersen’s model examined factors influencing colorectal cancer screening in the United States (US) and found that both men and women had comparable screening rates. For women, higher screening rates were associated with age, marital status, ethnicity, and education beyond bachelor’s degree. For men, influential factors included age, education beyond high school, and military insurance coverage. Being a migrant or a recent US resident was associated with reduced screening rates for both groups [20]. Another study analyzed factors influencing adherence to breast cancer screening guidelines among 5,484 female participants. Key findings revealed disparities in screening, especially among uninsured or financially constrained women in rural areas, suggesting the need for policy changes to improve healthcare access [21]. A different national US study examined the utilization of recommended preventive screenings and pinpointed several sociodemographic factors influencing engagement in preventive CVD screenings. Results showed that women were more likely to have their blood glucose levels checked, and that people with at least secondary education underwent lipid tests more often. The authors concluded that their findings should inform efforts to optimize recommended services [22]. In Australia, a qualitative study surveyed parents to explore the adoption of preventive services for their young children. The findings highlighted the need for increased flexibility and access to services, along with the importance of strengthening interprofessional relationships of healthcare providers [23]. The findings from a 2018 study from China focusing on the correlation between education level and preventive care utilization suggested that individuals with higher education levels had better access to preventive care than those with primary or no education. The study emphasized the need for targeted educational programs on the importance of preventive care [24]. Locally, few studies have utilized a similar model. One study examined the utilization of dental services by adults using secondary data from Saudi Arabia’s World Health Survey 2019 and found that key predictors of dental visits included income and free access to governmental healthcare services [25].

The novelty of our study lies in addressing the scarcity of evidence on preventive healthcare services in Saudi Arabia, particularly in cardiovascular prevention, which carries a significant public health burden.

## Materials and methods

### Study design

This analytical cross-sectional study measured both independent (predisposing, enabling, and need factors) and dependent (use of service) factors simultaneously using a validated self-administered questionnaire completed by individuals visiting a PHC during the data collection period.

### Study setting

This study was conducted at Al-Wazarat Healthcare Center (WHC) in Riyadh, the principal PHC under PSMMC. PSMMC operates within the Armed Forces Health Services (AFHS), which is governed by the Ministry of Defense [16]. We chose WHC for several reasons: *i)* WHC is the largest PHC under PSMMC administration; it is also the nearest to the PSMMC main campus. The size and central location of WHC facilitated access for visitors. *ii)* The center provides care to a diverse population across Riyadh. The center has the highest flow of visitors when compared to other multiple PHCs under PSMMC, making it an optimal catchment area for obtaining a large and diverse sample, enhancing the external validity of the study. *iii)* The center encompasses both general and specialized clinics, along with radiology and laboratory services, thereby providing comprehensive access to PCCS in one location. *iv)* Additionally, WHC is part of a program aimed at enhancing the quality and delivery of primary care centers under AFHS. All these factors made WHC an ideal location for conducting this study.

Our study targeted visitors seeking elective medical care at the General Family Medicine Clinics at WHC. These clinics provide comprehensive preventive and curative services to all individuals across all age groups and serves as the primary healthcare facility utilized by the visitors. Appointments can be scheduled by any visitor using an online portal, a mobile app, or through phone calls. Access to General Family Medicine Clinics is limited to individuals who have scheduled an appointment, whether for a new consultation or a follow-up. Each day, a predetermined number of appointment slots are opened for new consultations, allowing new visitors to schedule an appointment within a three-day timeframe.

### Data collection period

We gathered our data over a period that spanned 5 weeks and 3 days, starting from February 18, 2024, to March 27, 2024.

### Study population

Target population comprised of adults, aged 18 years and older, seeking elective medical care at a primary care setting under AFHS. The study population specifically included adults aged 18 years and above who sought elective medical care through appointments at the General Family Medicine Clinics at WHC during the data collection period.

Since most PCCS target adult individuals, we excluded individuals <18 years. Individuals who declined participation or opted not to complete the questionnaire for any reason were excluded from the study. Additionally, responses from individuals who did not complete the questionnaire were excluded.

### Sample size

The required sample size was determined using the open-source Raosoft Sample Size Calculator (2004, Raosoft, Inc., Seattle, USA) based on a confidence level of 95% and a margin of error set at 5%. The population size was based on the number of active electronic medical records which exceeded 1,200,000 at the time of the study. This calculation determined that a sample size of 384 respondents was required. To account for potential non-responses or incomplete data, we increased the sample size by an additional 10%. We achieved a response rate of 90% consistent with studies conducted in the same settings in Saudi Arabia.

### Sampling technique

Respondents were chosen using convenience sampling technique since *i)* the complete sampling frame remained inaccessible throughout the data collection period, as appointment slots were made available daily; *ii)* appointments are scheduled based on visitors’ preferences and do not adhere to any stringent eligibility criteria, enhancing the limited representativeness of this technique.

To mitigate this, the study was conducted at the largest PHC and spanned a period of 5 weeks across different days and work shifts, covering diverse demographics and reasons for visits to reduce potential selection bias.

### Data collection instrument

Data were collected using a printed questionnaire available in both Arabic and English, designed to capture two objectives: Andersen’s model components and PCCS use. We added three screening questions. *i)* Did you have an appointment at the General Family Medicine Clinics at this center today? *ii)* Have you ever visited a General Family Medicine Clinic at this center? *iii)* Have you ever been diagnosed with any cardiovascular disease? Those who answered “no” to questions *i* and *ii* were excluded. A trained nurse measured and recorded the exact weight and height for each respondent to calculate Body Mass Index (BMI).

To ensure content validity, the original questionnaire was developed based on extensive literature review and was subsequently reviewed by three experts from the Preventive Medicine Division and Family and Community Medicine Department at PSMMC. Additionally, a pretest of the questionnaire was conducted to ensure clarity and accuracy in capturing the intended data. Using convenience sampling, the pretest involved 10% of the sample size from the target population, conducted at a different PHC under PSMMC administration. The pretesting sample followed the same implied consent process, whereby proceeding to the questionnaire indicated agreement to participate. The results of the pretest were not included in the final analysis but were used to refine the questionnaire and calculate reliability using Cronbach’s Alpha, which yielded 0.737, indicating acceptable reliability.

Finally, following the initial translation into Arabic, back-translation into English was performed by a different bilingual expert who was blind to the original questionnaire. Respondents were offered either an Arabic or English questionnaire based on their preference. A version of the data collection tool in both languages is provided in S1 Appendix.

### Explanatory variables

#### Predisposing, enabling and need factors.

Factors under each component of Andersen’s model were drawn from previous studies [20, 25, 26], with the exclusion of contextual factors proposed by the model as they are beyond the scope of this research (e.g., social composition, community norms and values). Nine socio-demographic variables constituted the predisposing factors domain of Andersen’s model. These include sex, age, nationality, highest level of education, current marital status, number of children (if applicable), current place of residence (inside Riyadh or to specify if outside Riyadh), current employment status, and average household monthly income. Enabling factors are defined as financial and organizational elements that facilitate access to healthcare [13]. These include assessing the presence of social support, ease of scheduling appointments at WHC, having regular access to a vehicle for transportation, the proximity of the nearest PHC, time spent in waiting rooms, facing any language barriers, and satisfaction with quality of care. Finally, need factors which explain individuals’ perceived needs for healthcare services and healthcare providers’ (HCPs) evaluated needs [13]. Need factors included having a first-degree family history of CVD, presence of high blood pressure or diabetes or high blood cholesterol, and self-rated health status on a Likert 5-point-scale from excellent to poor.

#### Use of PCCS.

The Binary “Yes/No” questions about PCCS were based on the latest national and international guidelines. Both the Saudi Public Health Authority (PHA) Clinical Preventive Guidelines (Third Edition, 2023) [27] and the United States Preventive Services Task Force (USPSTF) recommendations [28–34] focus on the prevention of cardiovascular diseases in three categories (screenings, counseling, and preventive medications) mainly in adults. Nine preventive cardiovascular services were assessed: *i)* Screening for hypertension, prediabetes (PreDM) and type 2 diabetes mellitus (T2DM), Abdominal Aortic Aneurysm (AAA), and waist-circumference for obesity; *ii)* counseling for weight-loss, healthy diet and physical activity, and smoking-cessation; and *iii)* use of statins for the primary prevention of CVD. Only interventions categorized as grades ‘A’ or ‘B’ were included as they signify the highest level of evidence and are universally applicable to all intended populations, unlike grade ‘C’. These guidelines were developed to guide clinicians in incorporating preventive services into their practice. Details about each recommendation are provided in Table 1. The inclusion criteria for each recommendation were formulated to encompass the largest group possible. For instance, the interval for blood-pressure screening is set at five years rather than annually. We assumed this extended screening frequency would enhance our group classification and reduce potential recall bias.

**Table 1:** Operational Definitions of Preventive Cardiovascular Clinical Services.

Topic	Guidelines & Grade	Recommendation	Frequency
**Screening for Hypertension in Adults**	**USPSTF** ^**1**^ *2021, grade A*	Both recommend screening asymptomatic adults ≥18 years.	Every 3–5 years for adults aged 18–39 years, and yearly for adults ≥40 years.
	**PHA** ^**2**^ *2023, grade A*		
**Screening for Pre-diabetes and Type 2 Diabetes in Adults**	**USPSTF** *2021, grade B*	Screen asymptomatic adults aged 35–70 years who are overweight (BMI^3^ ≥ 25 kg/m^2^) or obese (BMI ≥ 30 kg/m^2^).	Every 3 years for adults with normal blood glucose levels.
	**PHA** *2023, grade B*	Screen using fasting blood sugar or hemoglobin A1C or random blood sugar starting at the age of 35 years. Screen all adults who are overweight and have additional risk factors^4^.	
**Screening for Abdominal Aortic Aneurysm**	**USPSTF** *2019, grade B*	Both recommend screening for AAA with ultrasonography in men aged 65–75 years who have ever smoked.	One-time.
	**PHA** *2023, grade B*		
**Screening of Waist Circumference for Obesity in Adults**	**PHA** *2023, ungraded*	Screen for BMI and waist circumference in all adults ≥18 years.	Every 2 years.
**Weight-Loss Counseling to Prevent Obesity-Related Morbidity and Mortality in Adults**	**USPSTF** *2018, grade B*	Both recommend offering or referring adults ≥18 years with BMI ≥ 30 kg/m^2^ to intensive, multicomponent behavioral interventions.	When risk factors are identified.
	**PHA** *2023, grade B*		Screen for BMI every 2 years.
**Counseling for Healthy Diet and Physical Activity for CVD Prevention in Adults with Cardiovascular Risk Factors**	**USPSTF** *2020, grade B*	Offer or refer adults ≥18 years with cardiovascular disease risk factors^5^ to behavioral counseling interventions to promote a healthy diet and physical activity.	When risk factors are identified.
**Tobacco Smoking-Cessation Counseling in Adults: Interventions**	**USPSTF** *2021, grade A*	Counsel all adults ≥18 years who use tobacco about cessation, provide behavioral interventions and approved pharmacotherapy (unless pregnant persons) for cessation.	When smoking is identified.
	**PHA** *2023, grade A*	Screen and counsel all adults ≥18 years who smoke about smoking-cessation.	
**Use of Statins for the Primary Prevention of Cardiovascular Disease in Adults**	**USPSTF** *2022, grade B*	Both recommend that clinicians prescribe a statin for the primary prevention of CVD for adults aged 40–75 years who have 1 or more CVD risk factors^6^ and an estimated 10-year risk of a cardiovascular event of 10% or greater.	When risk factors are identified. Screen for fasting lipid profile every 5 years*
	**PHA** *2023, grade A*		

^1^**USPSTF:** United States Preventive Services Task Force.

^2^**PHA:** Saudi Public Health Authority.

^3^**BMI**: Body Mass Index (kg/m^2^)

^4^**Additional risk factors to screen for Pre-diabetes and Type 2 Diabetes under PHA’s recommendations include**: physical inactivity, first-degree relative with diabetes, women who delivered a baby >4 kg or were diagnosed with gestational diabetes mellitus, blood pressure ≥140/90 or on therapy for hypertension, HDL cholesterol level <35 mg/dl or Triglycerides level >250mg/dl, women with polycystic ovary syndrome, impaired glucose tolerance test or impaired fasting glucose, hemoglobin A1c ≥ 5.7%, or a history of CVD.

^5^**Additional risk factors to offer or refer for healthy diet and physical diet counseling include**: hypertension, dyslipidemia, mixed or multiple risk factors such as metabolic syndrome, or an estimated 10-year CVD risk of ≥7.5%.

^6^**Risk factors to prescribe statin for primary prevention of CVD include**: dyslipidemia, diabetes, hypertension, or smoking.

*Our justification to use 5 years as screening interval for lipid disorders is based on an outdated 2013 USPSTF recommendation ‘Lipid Disorders in Adults (Cholesterol, Dyslipidemia): Screening **[35]**’. Both latest USPSTF and PHA recommendations do not specify a period of screening, while PHA recommends screening using fasting lipids profile with no details.

### Data analysis

Descriptive statistics were employed to summarize the predisposing factors and the utilization of PCCS among two groups of respondents: *i)* at-risk respondents who received the service (ARR), and *ii)* at-risk respondents who did not receive the service (ARDR). This involved calculating frequencies and percentages along with PCCS utilization profiles.

To examine the associations between PCCS and predisposing, enabling, and need factors, we conducted a simple logistic regression analysis. This method allowed us to estimate a crude odds ratio and 95% confidence intervals (CI) for each factor. Later, a multivariable logistic regression analysis was employed to develop a predictive model for each of the selected PCCS. A forward selection method was employed to identify the most significant independent variables within Andersen’s model. The modeling process began with the inclusion of the strongest predictors related to the dependent factor, followed by the sequential addition of variables until the remaining predictors were larger than *p = 0.05*. This method allowed for the exploration of the most parsimonious model that adequately describes the relationship between predictor variables and the outcome. Model fit was evaluated using the Chi-square goodness-of-fit and Nagelkerke R^2^ statistics. All analyses were performed using IBM SPSS Statistics version 25 (SPSS Inc, Chicago, Ill, USA). The results were interpreted with a significance level set at *p* ≤ 0.05.

### Ethical considerations

Ethical approval for this study was obtained from the Institutional Review Board of PSMMC (Approval No. E-2274). An informed consent form was provided as the first page of the questionnaire. The form clearly stated the study’s purpose, types of data to be collected, along with participants’ full rights to withdraw from the study at any time with no consequences. Participants were informed that by proceeding to answer the questionnaire, they were providing informed consent. In addition, trained data collectors verbally reiterated this to the participants. Confidentiality was ensured as no personal identifiers were collected, and access to raw data was limited to research team only. All results are reported solely in aggregate forms.

## Results

### Respondents’ demographics

The study included 384 respondents, all residing within Riyadh, with a predominance of female individuals (58%) and Saudi nationals (88%). The average age of respondents was 47.1 years (SD = 13.4, range 19–88 years). The majority of respondents received some form of formal education (88%). The majority were married at the time of the study (64%), and 74% had at least one child, including those previously married.

Screening questions revealed that 53% of respondents were known CVD patients, 46% were smokers or were regularly exposed second-hand smoking, and 74% were overweight or obese. (See Table 2)

**Table 2:** Predisposing Factors for Respondents seen at General Family Medicine Clinics at WHC, PSMMC, Riyadh, Saudi Arabia (N = 384).

Characteristics		n (%)
**Sex**	Male	162 (42.2)
	Female	222 (57.8)
**Age**	18-34 years	88 (22.9)
	35-44 years	70 (18.2)
	45-54 years	85 (22.1)
	55-64 years	102 (26.6)
	65 + years	39 (10.2)
**Nationality**	Saudi	337 (87.8)
	Non-Saudi	47 (12.2)
**Highest education received**	Non-formal education	47 (12.2)
	High-school or lower	219 (57.0)
	Bachelor or higher	118 (30.8)
**Current marital status**	Married	245 (63.8)
	Unmarried	139 (36.2)
**Has children**	No	100 (26)
	Yes	284 (74)
**Current employment status**	Student	10 (2.6)
	Unemployed	80 (20.8)
	Employed at any sector	195 (50.8)
	Self-employed	88 (22.9)
	Retired	11 (2.9)
**Household monthly income**	<5,000 SAR^1^	13 (3.4)
	5,000–10,000 SAR	24 (6.3)
	10,000–15,000 SAR	84 (21.9)
	≥15,000 SAR	263 (68.4)
**Known cardiovascular disease**	No	180 (46.9)
	Yes	204 (53.1)
**Current smoking status**	Smoker or exposed to smoking	177 (46.1)
	Non-smoker	207 (53.9)
**BMI**^**2**^ **categories**	Normal weight	98 (25.5)
	Underweight	1 (0.3)
	Overweight	190 (49.5)
	Obese	95 (24.7)

^1^SAR: Saudi Arabia Riyals; ^2^BMI: Body Mass Index

### Overall Utilization of PCCS

The utilization among study respondents varied across services based on PHA’s and USPSTF’s guidelines risk categories.

While screening for hypertension was utilized by 93% of individuals at risk (i.e., those aged ≥18 years), waist-circumference screening was utilized by 16% of participants, and nearly half of those eligible for obesity counseling did not receive it (49%). Smoking-cessation counseling was not offered to 60% of individuals who smoked, while AAA screening in males aged ≥65 was offered to only one respondent. Table 3 explains the utilization variation between individuals at risk who received or did not receive the recommended service.

**Table 3:** PCCS Utilization Rates among At-risk Respondents seen at General Family Medicine Clinics at WHC, PSMMC, Riyadh, Saudi Arabia.

Utilized Service (N)	ARR^1^ n (%)	ARDR^2^ n (%)
**Screening for Hypertension (384)**	356 (93)	28 (7)
Screening for Pre-diabetes and Type 2 Diabetes (284) *	246 (87)	38 (13)
Screening of Waist Circumference for Obesity (384) *	61 (16)	323 (84)
Use of Statins as Preventive Medication (209) *	181 (87)	28 (13)
**Screening for AAA**^**3**^ among smokers (25) *	1 (4)	24 (96)
Counseling for Weight-Loss in Adults (96) *	49 (51)	47 (49)
Counseling for Healthy Diet (223) *	173 (78)	50 (22)
Counseling for Physical Activity (223) *	188 (84)	35 (16)
Counseling for Tobacco Smoking-Cessation (177) *	71 (40)	106 (60)

^1^ARR: At-risk respondents who received the service, ^2^ARDR: At-risk respondents who did not receive the service;

^3^AAA: Abdominal Aortic Aneurysm;

*The actual number of the individuals targeted with the service out of 384 survey respondents.

### Factors Influencing the Utilization of PCCS

Results for PCCS related to screenings and preventive medications are summarized in Table 4, while services related to counseling are summarized in Table 5. Results for AAA screening are presented in text. In this section, we report the significance levels for crude OR (COR).

**Table 4:** Utilization of Preventive Cardiovascular Screening and Preventive Medications Services according to Andersen’s Model of among Respondents seen at General Family Medicine Clinics at WHC, PSMMC, Riyadh, Saudi Arabia.

Characteristics			Hypertension Screening			PreDM/T2DM^1^ Screening			Waist-Circumference Screening			Statin Use		
			ARDR^2^ n (%)	ARR^3^ n (%)	*P-value*	ARDR n (%)	ARR n (%)	*P-value*	ARDR n (%)	ARR n (%)	*P-value*	ARDR n (%)	ARR n (%)	*P-value*
**Predisposing Factors**	**Sex**	Male*	9 (5.6)	153 (94.4)	0.267	9 (8.2)	101 (91.8)	**0.045**	150 (92.6)	12 (7.4)	**<0.001**	20 (17.5)	94 (82.5)	0.059
												8 (8.4)	87 (91.6)	
		Female	19 (8.6)	203 (91.4)		29 (16.7)	145 (83.3)		173 (77.9)	49 (22.1)				
	**Age**	18-34 years*	23 (26.1)	65 (73.9)	**<0.001**	16 (37.2)	27 (62.8)	**<0.001**	84 (95.5)	4 (4.5)	**0.002**	–	–	**0.001**
		35-44 years	3 (4.3)	67 (95.7)		12 (23.1)	40 (76.9)		63 (90)	7 (10)		–	–	
		45-54 years	2 (2.4)	83 (97.6)		6 (9.0)	61 (91.0)		69 (81.2)	16 (18.8)		19 (26.4)	53 (73.6)	
		55-64 years	0 (0)	102 (100)		3 (3.5)	83 (96.5)		80 (78.4)	22 (21.6)		7 (7.1)	91 (92.9)	
		65 + years	0 (0)	39 (100)		1 (2.8)	35 (97.2)		27 (69.2)	12 (30.8)		2 (5.1)	37 (94.9)	
	**Nationality**	Non-Saudi*	3 (6.4)	44 (93.6)	0.798	3 (8.6)	32 (91.4)	0.378	41 (87.2)	6 (12.8)	0.533	3 (21.4)	11 (78.6)	0.368
		Saudi	25 (7.4)	312 (92.6)		35 (14.1)	214 (85.9)		282 (83.7)	55 (16.3)		25 (12.8)	170 (87.2)	
	**Highest Education Received**	Non formal *	0 (0)	47 (100)	0.664	2 (4.5)	42 (95.5)	0.086	27 (57.4)	20 (42.6)	**<0.001**	3 (6.8)	41 (93.2)	0.259
		High-school or less	16 (7.3)	203 (92.7)		22 (13.0)	147 (87.0)		187 (85.4)	32 (14.6)		20 (14.2)	121 (85.8)	
						14 (19.7)	57 (80.3)		109 (92.4)	9 (7.6)		5 (20.8)	19 (79.2)	
		Bachelors’ or higher	12 (10.2)	106 (89.8)										
	**Current Marital Status**	Unmarried*	16 (11.5)	123 (88.5)	**0.020**	18 (19.8)	73 (80.2)	**0.032**	121 (87.1)	18 (12.9)	0.237	10 (17.2)	48 (82.8)	0.314
		Married	12 (4.9)	233 (95.1)		20 (10.4)	173 (89.6)		202 (82.4)	43 (17.6)		18 (11.9)	133 (88.1)	
	**Has Children**	No*	20 (20)	80 (80)	**<0.001**	24 (40)	36 (60)	**<0.001**	93 (93)	7 (7)	**0.007**	6 (37.5)	10 (62.5)	**0.006**
		Yes	8 (2.8)	276 (97.2)		14 (6.3)	210 (93.8)		230 (81)	54 (19)		22 (11.4)	171 (88.6)	
	**Current Employment Status**	Unemployed*	7 (6.9)	94 (93.1)	0.871	5 (6.3)	75 (93.8)	**0.033**	72 (71.3)	29 (28.7)	**<0.001**	7 (9.1)	70 (90.9)	0.168
		Employed	21 (7.4)	262 (92.6)		33 (16.2)	171 (83.8)		251 (88.7)	32 (11.3)		21 (15.9)	111 (84.1)	
	**Household Monthly Income**	<15000 SAR*	10 (8.3)	111 (91.7)	0.619	10 (10.2)	88 (89.8)	0.257	95 (78.5)	26 (21.5)	**0.043**	6 (10)	54 (90)	0.363
		≥15000 SAR	18 (6.8)	245 (93.2)		28 (15.1)	158 (84.9)		228 (86.7)	35 (13.3)		22 (14.8)	127 (85.2)	
**Enabling Factors**	**Social Support**	No*	2 (20)	8 (80)	0.139	1 (11.1)	8 (88.9)	0.839	7 (70)	3 (30)	0.229	1 (14.3)	6 (85.7)	0.944
		Yes	26 (7)	348 (93)		37 (13.5)	238 (86.5)		316 (84.5)	58 (15.5)		27 (13.4)	175 (86.6)	
	**Easy Booking**	No*	7 (9.3)	68 (90.7)	0.450	11 (19.3)	46 (80.7)	0.146	60 (80)	15 (20)	0.279	5 (13.5)	32 (86.5)	0.982
		Yes	21 (6.8)	288 (93.2)		27 (11.9)	200 (88.1)		263 (85.1)	46 (14.9)		23 (13.4)	149 (86.6)	
	**Owns a Vehicle**	No*	7 (5)	132 (95)	0.205	17 (15.6)	92 (84.4)	0.388	118 (84.9)	21 (15.1)	0.754	13 (16.5)	66 (83.5)	0.314
		Yes	21 (8.6)	224 (91.4)		21 (12)	154 (88)		205 (83.7)	40 (16.3)		15 (11.5)	115 (88.5)	
	**Time Travel to Nearest PHC.**	≥1 hour*	6 (4.1)	142 (95.9)	0.060	11 (10.3)	96 (89.7)	0.236	118 (79.7)	30 (20.3)	0.064	14 (15.4)	77 (84.6)	0.460
		<1 hour	22 (9.3)	214 (90.7)		27 (15.3)	150 (84.7)		205 (86.9)	31 (13.1)		14 (11.9)	104 (88.1)	
	**Waiting Time**	≥30 minutes*	23 (7.4)	287 (92.6)	0.844	35 (14.9)	200 (85.1)	0.113	258 (83.2)	52 (16.8)	0.332	24 (13.3)	157 (86.7)	0.882
		<30 minutes	5 (6.8)	69 (93.2)		3 (6.1)	46 (93.9)		65 (87.8)	9 (12.2)		4 (14.3)	24 (85.7)	
	**Language Barriers**	Yes*	1 (1.3)	74 (98.7)	0.057	4 (6.1)	62 (93.9)	0.055	56 (74.7)	19 (25.3)	**0.014**	6 (11.1)	48 (88.9)	0.568
		No	27 (8.7)	282 (91.3)		34 (15.6)	184 (84.4)		267 (86.4)	42 (13.6)		22 (14.2)	133 (85.8)	
	**Perceived Quality of Care**	No*	2 (6.7)	28 (93.3)	0.891	4 (14.3)	24 (85.7)	0.882	24 (80)	6 (20) 55	0.522	1 (6.7)	14 (93.3)	0.439
		Yes	26 (7.3)	328 (92.7)		34 (13.3)	222 (86.7)		299 (84.5)	(15.5)		27 (13.9)	167 (86.1)	
**Need Factors**	**1**^**st**^ **Degree Family History Of CVD**	No*	8 (21.6)	29 (78.4)	**0.001**	9 (34.6)	17 (65.4)	**0.002**	34 (91.9)	3 (8.1)	0.184	3 (25)	9 (75)	0.236
		Yes	20 (5.8)	327 (94.2)		29 (11.2)	229 (88.8)		289 (83.3)	58 (16.7)		25 (12.7)	172 (87.3)	
	Presence of CVD Risk Factors*	No*	26 (16.1)	135 (83.9)	**<0.001**	32 (33)	65 (67.0)	**<0.001**	150 (93.2)	11 (6.8)	**<0.001**	4 (30.8)	9 (69.2)	0.070
		Yes	2 (0.9)	221 (99.1)		6 (3.2)	181 (96.8)		173 (77.6)	50 (22.4)		24 (12.2)	172 (87.8)	
	**Perceived Health Status**	Poor*	1 (2.6)	38 (97.4)	**<0.001**	0 (0)	34 (100)	**0.001**	27 (69.2)	12 (30.8)	**0.001**	1 (3)	32 (97)	0.108
		Average	3 (1.7)	175 (98.3)		11 (7.5)	135 (92.5)		143 (80.3)	35 (19.7)		24 (17)	117 (83)	
		Excellent	24 (14.4)	143 (85.6)		27 (26.0)	77 (74.0)		153 (91.6)	14 (8.4)		3 (8.6)	32 (91.4)	

^1^PreDM/T2DM: Pre-diabetes, Type 2 diabetes mellitus; ^2^ARDR: At-risk respondents who did not receive service; ^3^ARR: At-risk respondents who received service.

*Reference value.

**Table 5:** Utilization of Preventive Cardiovascular Counseling Services according to Andersen’s Model among Respondents seen at General Family Medicine Clinics at WHC, PSMMC, Riyadh, Saudi Arabia.

Characteristics			Weight-Loss Counseling			Healthy-Diet Counseling			Physical Activity Counseling			Smoking-Cessation Counseling		
			ARDR^1^ n (%)	ARR^2^ n (%)	*P-value*	ARDR n (%)	ARR n (%)	*P-value*	ARDR n (%)	ARR n (%)	*P-value*	ARDR n (%)	ARR n (%)	*P-value*
**Predisposing Factors**	**Sex**	Male*	18 (72)	7 (28)	**0.009**	34 (29.8)	80 (70.2)	**0.008**	26 (22.8)	88 (77.2)	**0.004**	58 (47.5)	64 (52.5)	**<0.001**
		Female	29 (40.8)	42 (59.2)		16 (14.7)	93 (85.3)		9 (8.3)	100 (91.7)		48 (87.3)	7 (12.7)	
	**Age**	18-34 years*	8 (88.9)	1 (11.1)	**0.039**	1 (20)	4 (80)	1.000	0 (0)	5 (100)	0.998	20 (74.1)	7 (25.9)	0.434
		35-44 years	8 (66.7)	4 (33.3)		5 (22.7)	17 (77.3)		4 (18.2)	18 (81.8)		16 (66.7)	8 (33.3)	
		45-54 years	12 (54.5)	10 (45.5)		14 (23)	47 (77)		10 (16.4)	51 (83.6)		26 (57.8)	8 (33.3)	
		55-64 years	13 (41.9)	18 (58.1)		22 (22.4)	76 (77.6)		10 (16.4)	83 (84.7)		30 (53.6)	26 (46.4)	
		65 + years	6 (27.3)	16 (72.7)		8 (21.6)	29 (78.4)		6 (16.2)	31 (83.8)		14 (56)	11 (44)	
	**Nationality**	Non-Saudi*	7 (77.8)	2 (22.2)	0.089	2 (10)	18 (90)	0.179	1 (5)	19 (95)	0.199	7 (50)	7 (50)	0.434
		Saudi	40 (46)	47 (54)		48 (23.6)	155 (76.4)		34 (16.7)	169 (83.3)		99 (60.7)	64 (39.3)	
	**Highest Education Received**	Non formal *	7 (25)	21 (75)	**0.002**	9 (20)	36 (80)	0.357	7 (15.6)	38 (84.4)	0.130	12 (60	8 (40	0.810
		High-school or less	26 (51)	25 (49)		36 (25.2)	107 (74.8)		27 (18.9)	116 (81.1)		72 (58.5)	51 (41.5)	
		Bachelors’ or higher	14 (82.4)	3 (17.6)		5 (14.3)	30 (85.7)		1 (2.9)	34 (97.1)		22 (64.7)	12 (35.3)	
	**Current Marital Status**	Unmarried*	13 (40.6)	19 (59.4)	0.250	14 (23.3)	46 (76.7)	0.843	11 (18.3)	49 (81.7)	0.512	32 (68.1)	15 (31.9)	0.183
		Married	34 (53.1)	30 (46.9)		36 (22.1)	127 (77.9)		24 (14.7)	139 (85.3)		74 (56.9)	56 (43.1)	
	**Has Children**	No*	6 (54.5)	5 (45.5)	0.694	4 (21.1)	15 (78.9)	0.881	2 (10.5)	17 (89.5)	0.521	22 (71)	9 (29)	0.170
		Yes	41 (48.2)	44 (51.8)		46 (22.5)	158 (77.5)		33 (16.2)	171 (83.8)		84 (57.5)	62 (42.5)	
	**Current Employment Status**	Unemployed*	14 (31.1)	31 (68.9)	**0.001**	13 (16.5)	66 (83.5)	0.116	8 (10.1)	71 (89.9)	0.095	26 (61.9)	16 (38.1)	0.760
		Employed	33 (64.7)	18 (35.3)		37 (25.7)	107 (74.3)		27 (18.8)	117 (81.3)		80 (59.3)	55 (40.7)	
	**Household Monthly Income**	<15000 SAR*	24 (49)	25 (51)	0.997	8 (11.6)	61 (88.4)	**0.012**	4 (5.8)	65 (94.2)	**0.011**	27 (65.9)	14 (34.1)	0.375
		≥15000 SAR	23 (48.9)	24 (51.1)		42 (27.3)	112 (72.7)		31 (20.1)	123 (79.9)		79 (58.1)	57 (41.9)	
**Enabling Factors**	**Social Support**	No*	1 (20)	4 (80)	0.216	1 (12.5)	7 (87.5)	0.502	0 (0)	8 (100)	0.999	3 (75.0)	1 (25.0)	0.541
		Yes	46 (50.5)	45 (49.5)		49 (22.8)	166 (77.2)		35 (16.3)	180 (83.7)		103 (59.5)	70 (40.5)	
	**Easy Booking**	No*	19 (67.9)	9 (32.1)	**0.020**	11 (28.2)	28 (71.8)	0.342	7 (17.9)	32 (82.1)	0.671	18 (52.9)	16 (47.1)	0.359
		Yes	28 (41.2)	40 (58.8)		39 (21.2)	145 (78.8)		28 (15.2)	156 (84.8)		88 (61.5)	55 (38.5)	
	**Owns a Vehicle**	No*	16 (39)	25 (61)	0.095	14 (16.1)	73 (83.9)	0.072	6 (6.9)	81 (93.1)	**0.006**	35 (76.1)	11 (23.9)	**0.011**
		Yes	31 (56.4)	24 (43.6)		36 (26.5)	100 (73.5)		29 (21.3)	107 (78.7)		71 (54.2)	60 (45.8)	
	**Time Travel to Nearest PHC.**	≥1 hour*	19 (45.2)	23 (54.8)	0.520	22 (22.9)	74 (77.1)	0.878	17 (17.7)	79 (82.3)	0.473	41 (54.7)	34 (45.3)	0.225
		<1 hour	28 (51.9)	26 (48.1)		28 (22)	99 (78)		18 (14.2)	109 (85.8)		65 (63.7)	37 (36.3)	
	**Waiting Time**	≥30 minutes*	39 (48.1)	42 (51.9)	0.712	41 (21.8)	147 (78.2)	0.611	29 (15.4)	159 (84.6)	0.798	89 (58.6)	63 (41.4)	0.374
												17 (68.0)	8 (32.0)	
		<30 minutes	8 (53.3)	7 (46.7)		9 (25.7)	26 (74.3)		6 (17.1)	29 (82.9)				
	**Language Barriers**	Yes*	9 (33.3)	18 (66.7)	0.059	15 (25.9)	43 (74.1)	0.466	9 (15.5)	49 (84.5)	0.965	22 (55.0)	18 (45.0)	0.474
		No	38 (55.1)	31 (44.9)		35 (21.2)	130 (78.8)		26 (15.8)	139 (84.2)		84 (61.3)	53 (38.7)	
	**Perceived Quality of Care**	No*	9 (69.2)	4 (30.8)	0.126	7 (41.2)	10 (58.8)	0.061	3 (17.6)	14 (82.4)	0.818	5 (50.0)	5 (50.0)	0.514
		Yes	38 (45.8)	45 (54.2)		43 (20.9)	163 (79.1)		32 (15.5)	174 (84.5)		101 (60.5)	66 (39.5)	
**Need Factors**	**1**^**st**^ **Degree Family History of CVD**	No*	5 (62.5)	3 (37.5)	0.429	2 (16.7)	10 (83.3)	0.625	1 (8.3)	11 (91.7)	0.481	8 (72.7)	3 (27.3)	0.376
		Yes	42 (47.7)	46 (52.3)		48 (22.7)	163 (77.3)		34 (16.1)	177 (83.9)		98 (59.0)	68 (41.0)	
	Presence of CVD Risk Factors*	No*	16 (84.2)	3 (15.8)	**0.002**	–	–	**<0.001**	–	–	**<0.001**	37 (72.5)	14 (27.5)	**0.031**
		Yes	31 (40.3)	46 (59.7)		50 (22.4)	173 (77.6)		35 (15.7)	188 (84.3)		69 (54.8)	57 (45.2)	
	**Perceived Health Status**	Poor*	2 (11.8)	15 (88.2)	**0.003**	8 (21.1)	30 (78.9)	0.451	6 (15.8)	32 (84.2)	0.938	8 (44.4)	10 (55.6)	**0.037**
		Average	25 (49)	26 (51)		30 (20.7)	115 (79.3)		22 (15.2)	123 (84.8)		57 (55.3)	46 (44.7)	
		Excellent	20 (71.4)	8 (28.6)		12 (30)	28 (70)		7 (17.5)	33 (82.5)		41 (73.2)	15 (26.8)	

^1^ARDR: At-risk respondents who did not receive service; ^2^ARR: At-risk respondents who received service.

#### Screening for hypertension.

Analysis of predisposing factors revealed a significant increase in screening with age (p ≤ 0.001). Married individuals and individuals who have children saw significant increase in utilization (p < 0.020 and p < 0.001, respectively). While all enabling factors showed no statistical significance, all need factors were statistically significant (p ≤ 0.001). Individuals were more likely to receive blood-pressure screening if they had a family history of CVD, had one or more CVD risk factor, or perceived their health as poor (See Table 4).

#### Screening for prediabetes and type 2 diabetes.

Predisposing factors such as age and female sex have significantly influenced utilization. For age, rates progressively increased from 63% (18–34 years) to 97% (≥65 years). Married respondents were more likely to be screened compared to unmarried ones (p < 0.032). Parenthood and employment differed significantly between subgroup; employed individuals were more likely to utilize services (p < 0.033). Similar to hypertension screening, analysis of enabling factors revealed no statistically significant factors, while all three need factors were statistically significant (p ≤ 0.001) (See Table 4).

#### Screening for waist circumference for obesity.

Waist-circumferences screening had the greatest number of significant predisposing factors. Sex showed a significant difference, with male individuals exhibiting lower utilization rates compared to female individuals (p < 0.001). Older age, higher educational attainment, parenthood, and active employment status were all significant factors. Respondents with higher household monthly income had lower screening rates (13%) than those with lower income (22%) (p = 0.043). Examination of enabling factors identified language barriers as the only statistically significant factor; respondents facing barriers were more likely to be screened than those with no language barriers (p = 0.014). Finally, the analysis of need factors showed significant associations among individuals with CVD risk factors or those who perceived their health as poor (p < 0.001 and p = 0.001, respectively) (See Table 4).

#### Use of statins for the primary prevention of CVD.

Analysis of predisposing factors highlighted significant variation in the utilization based on age (p = 0.001) and parenthood status (p = 0.006). Statins were offered more frequently in older age groups, with utilization rates reaching 74% (35–44 years) and 95% (≥65 years). Enabling and need factors showed no statistically significant differences (Table 4).

#### Weight-loss counseling to prevent obesity-related morbidity and mortality.

Predisposing factors analysis for weight-loss counseling identified significant differences in utilization based on sex (female individuals, p = 0.009), age (older adults, p = 0.039), education (no formal education, p = 0.002), and employment status (unemployed, p = 0.001).

Enabling factors analysis showed that respondents who found booking appointments easy were more likely to be offered counseling than those who found it difficult (p = 0.020). Among need factors, the presence of CVD risk factors significantly increased counseling utilization (p = 0.002). Additionally, individuals with poor perceived health were significantly more likely to receive counseling compared to those reporting excellent health (p = 0.003) (See Table 5).

Counseling for healthy diet and physical activity for cardiovascular prevention in adults with cardiovascular risk factors.

When analyzing healthy diet and physical activity separately, significant disparities were observed for two predisposing factors. Female individuals were significantly more likely to receive both healthy-diet (p = 0.008) and physical activity counseling (p = 0.004) compared to male individuals, and respondents earning <15000/month showed higher counseling utilization for both services. Notably, for enabling factors, owning a vehicle significantly impacted physical activity counseling (p = 0.006). Finally, the presence of one or more CVD risk factors significantly influenced utilization for both services (p < 0.001), with all recipients having at least one CVD risk factor (See Table 5).

#### Tobacco smoking-cessation counseling.

Analysis revealed a statistically significant difference by sex in the provision of counseling (p < 0.001), with 52% male respondents receiving counseling compared to 13% of female respondents. Among enabling factors, vehicle ownership emerged as significant; vehicle owners were more likely to participate in counseling than non-owners (p = 0.011). The presence of one or more CVD risk factors and poor perceived health status were significant need factors (p = 0.031 and p = 0.037, respectively) (See Table 5).

#### Screening for abdominal aortic aneurysm.

Due to small cell counts, Fisher’s exact test was performed to examine the association between risk group and receiving AAA screening. The results indicated that no single predisposing, enabling, or need factor had a significant association with screening utilization. Additionally, some variables, such as nationality, parenthood, and education were not analyzed due to the lack of variation in the sample.

### Predictors of PCCS utilization

Table 6 presents the models used to explore predictors for each service among study respondents. Each model identifies variables that act independently (COR) or in concert with one another (Adjusted OR; AOR) to explain variation in service utilization.

**Table 6:** Associations between Utilization Factors According to Andersen’s Model and Preventive Cardiovascular Clinical Services (PCCS) among Respondents seen at General Family Medicine Clinics at WHC, PSMMC, Riyadh, Saudi Arabia.

Service	Characteristics	Crude OR [95% CI]	*P*-value	Adjusted OR [95% CI]	*P-value*
**Model-1:** ***Hypertension Screening***	Age ≥ 35 years	20.6 [7.55, 56.2]	**<0.001**	22.1 [7.22, 67.5]	**<0.001**
	Socially supported	3.34 [0.67, 16.5]	0.139	19.05 [2.04, 178.3]	**0.010**
	Time traveled >1 hour	2.43 [0.96, 6.15]	**0.060**	3.07 [1.05-8.96]	**0.040**
	Having language barriers	7.08 [0.94, 52.9]	0.057	8.25 [0.96-70.7]	0.054
	Family history of CVD	4.51 [1.83, 11.1]	**<0.001**	4.87 [1.57, 15.2]	**0.006**
**Model-2:** ***PreDM/T2DM***^*1*^ ***Screening***	Has children	10 [4.73, 21.1]	**<0.001**	4.8 [2.00, 11.31]	**<0.001**
	Waiting time <30 min	2.7 [0.8, 9.1]	0.101	4.4 [1.21, 16.5]	**0.027**
	Has CVD risk factors	14.9 [5.94, 37.15]	**<0.001**	8.7 [3.23, 23.7]	**<0.001**
**Model-3:** ***Waist-Circumference Screening***	Female sex	3.54 [1.81, 6.91]	**<0.001**	9.24 [4.17, 20.45]	**<0.001**
	High school or less	2.94 [1.39, 6.19]	**0.004**	2.81 [1.22-6.50]	**0.015**
	Owning a vehicle	1.10 [0.62, 1.95]	0.754	2.88 [1.42, 5.83]	**0.003**
	Time traveled >1 hour	1.68 [0.96, 2.91]	0.064	2.20 [1.17-4.13]	**0.014**
	Has CVD risk factors	3.94 [1.98, 7.85]	**<0.001**	5.36 [2.47, 11.62]	**<0.001**
**Model-4:** ***Statin Use***	Female sex	2.31 [0.97, 5.52]	0.054	3.52 [1.34, 9.24]	**0.011**
	Has children	4.66 [1.54, 14.1]	**0.006**	6.14 [1.86, 20.3]	**0.003**
	Own a vehicle	1.51 [0.68, 3.37]	0.312	2.66 [1.07, 6.60]	**0.036**
**Model-5:** ***Weight-Loss Counseling***	Female sex	3.72 [1.38, 10.05]	**0.009**	6.56 [1.77, 24.3]	**0.005**
	Has no children	0.77 [0.22, 2.74]	0.694	5.33 [0.92, 30.6]	0.061
	High school or less	6.5 [1.73, 24.4]	**0.006**	6.20 [1.18, 32.4]	**0.031**
	Easy bookings	3.02 [1.19, 7.63]	**0.020**	4.69 [1.38, 15.9]	**0.013**
	Has CVD risk factors	7.91 [2.13, 29.4]	**0.002**	11.04 [1.99, 61.3]	**0.006**
	Poor health	9.92 [2.12, 46.3]	**0.004**	14.3 [2.07, 99.6]	**0.007**
**Model-6:** ***Healthy-Diet Counseling***	Female sex	2.47 [1.27, 4.80]	**0.008**	2.05 [1.03, 4.11]	**0.042**
	Income <15000 SAR	2.85 [1.26, 6.47]	**0.012**	2.05 [1.02, 4.10]	**0.034**
	Received quality care	2.65 [0.95, 7.38]	0.061	3.17 [1.08, 9.27]	**0.035**
**Model-7:** ***Physical Activity Counseling***	Female sex	3.28 [1.46, 7.38]	**0.004**	2.13 [0.89, 5.10]	0.089
	Bachelor’s or higher	7.51 [0.99, 56.8]	0.051	7.92 [1.03, 60.9]	**0.047**
	Owning no vehicle	3.65 [1.45, 9.22]	**0.006**	3.04 [1.13, 8.17]	**0.027**
**Model-8:** ***Smoking Counseling***	Male sex	7.56 [3.17, 18.0]	**<0.001**	8.94 [3.56, 22.4]	**<0.001**
	Excellent health	2.08 [0.75, 5.36]	0.165	3.44 [1.06, 11.1]	**0.038**

^1^PreDM/T2DM: Pre-diabetes, Type 2 diabetes mellitus

#### Model-1: Screening for hypertension.

The model was statistically significant (*x*^2^ (5) = 68.7, p < 0.001) explained up to 40% of the variance (Nagelkerke R square) and correctly classified 92.7% of cases. However, the Hosmer-Lemeshow test indicated poor fit, *x*^2^ (6) =64.49, p < 0.001). The model revealed that respondents aged ≥35 years (AOR = 22.1, p < 0.001), who were socially supported (AOR = 19, p = 0.010), traveled more than one hour to the nearest PHC (AOR = 3.07, p = 0.040), had language barriers (AOR = 8.2, p = 0.054), and had a positive family history of CVD (AOR = 4.8, p = 0.006) were more likely to utilize hypertension screening services.

#### Model-2: Screening for prediabetes and type 2 diabetes.

This model was also statistically significant (*x*^2^ (3) = 66.6, p < 0.001) explaining up to 38% of the variance in service use and correctly classifying 86.6% of cases, with a good fit to the data (*x*^2^ (3) =5.8, p = 0.119). This model shows that respondents who have children (AOR = 4.8, p < 0.001), waited <30 minutes in the waiting room (AOR = 4.4 p = 0.027), and had a CVD risk factor (AOR = 8.7, p < 0.001), were more likely to use PreDM and T2DM screenings.

#### Model-3: Screening for waist circumference for obesity.

Waist-circumference screening model was statistically significant (*x*^2^ (5) = 62.2, p < 0.001). The model explained up to 26% of the variance in screening utilization and correctly classified 84% of cases. Goodness-of-fit test suggested a good fit (*x*^2^ (8) =1.6, p = 0.990). Female respondents (AOR = 9.2, p < 0.001), who had a high school education or less (AOR = 2.8, p = 0.015), own a vehicle (AOR = 2.8, p = 0.003), traveled for more than one hour to the nearest PHC (AOR = 2.2, p = 0.014), and had at least one CVD risk factor (AOR = 5.3, p < 0.001) were more likely to utilize waist-circumference screening.

#### Model-4: Use of statins for the primary prevention of CVD.

This model correctly classified 86.6% of cases and was statistically significant (*x*^2^ (3) = 15.318, p = 0.002). However, it explained up to 13% of the variance using Nagelkerke R square, with a good fit to the data (*x*^2^ (4) =2.43, p = 0.656). The model shows that female respondents (AOR = 3.5, p = 0.011) who have children (AOR = 6.1, p = 0.003), and own a vehicle (AOR = 2.6, p = 0.036), were using more statin prescription services.

#### Model-5: Weight-loss counseling to prevent obesity-related morbidity and mortality.

This significant model (*x*^2^ (6) = 45.6, p < 0.001) correctly classified 51% of cases and was able to explain up to 50% of the variance and had a good fit to the data (*x*^2^ (5) =3.7, p = 0.594). The significant variables in this model included the following: female individuals (AOR = 6.5, p = 0.005), who have no children (AOR = 5.3, p = 0.061), with the highest educational attainment being high school degree or less (AOR = 6.2, p = 0.031), who found it easy to book an appointment (AOR = 4.6, p = 0.013), and had both CVD risk factors (AOR = 11, p = 0.006), and a reported health status as poor (AOR = 14.3, p = 0.007) were more likely to receive weight-loss counseling services.

#### Model-6: Counseling for healthy diet for cardiovascular prevention.

Healthy-diet counseling model was statistically significant (*x*^2^ (3) = 15.8, p = 0.001). The model explained up to 10% of the variance in counseling utilization and correctly classified 78% of cases. Goodness-of-fit test suggested a good fit (*x*^2^ (4) =1.9, p = 0.748). The model revealed that female individuals (AOR = 2.05, p = 0.042), with income <15,000 SAR (AOR = 2.05, p = 0.034), and who reported receiving high-quality care (AOR = 3.17, p = 0.035) were more likely to receive healthy-diet counseling.

#### Model-7: Counseling for physical activity for cardiovascular prevention.

The physical activity counseling model showed good fit (*x*^2^ (3) =1.5, p = 0.685), and was statistically significant (*x*^2^ (3) = 20.7, p < 0.001). The model explained up to 15% of the variance in counseling utilization and correctly classified 84% of cases. The model shows that female individuals (AOR = 2.1, p = 0.089), who have a bachelor’s degree or more (AOR = 7.9, p = 0.047), and did not own a vehicle (AOR = 3.04, p = 0.027) were more likely to be offered this counseling service.

#### Model-8: Tobacco smoking-cessation counseling.

The model was significant (*x*^2^ (2) = 32.2, p < 0.001), correctly classified 60% of cases, explained up to 23% of the variance, and showed good fit, (*x*^2^ (2) =0.082, p = 0.960). Male respondents (AOR = 8.94, p < 0.001), and those reporting poor health (AOR = 3.4, p = 0.038) were more likely to receive smoking-cessation counseling.

## Discussion

Using Andersen’s model as a framework, this study identified the factors influencing the utilization of PCCS provided under the AFHS primary healthcare setting. To establish a unified basis, this study examined PCCS in accordance with national and international clinical guidelines. In this study, we focused on individuals receiving the service rather than providers (i.e., HCPs) to understand their challenges. This approach considers the variability in knowledge and skills among HCPs.

This section specifically explores the influence of the dependent variables within each regression model (See Table 6).

### The high prevalence of CVD and its risk factors

First, the high prevalence of CVD among respondents (53%) underscores the critical need for a comprehensive cardiovascular prevention program across all levels of prevention within AFHS. Demographic data further reveal a high prevalence of multiple risk factors, such as smoking or exposure to second-hand smoke (46%), being overweight or obese (74%), self-reported diagnoses of hypertension, diabetes, or dyslipidemia (58%), or having a positive family history of CVD (90.4%). These factors, among others, are well-documented and extensively studied as contributors to CVD [6]. With CVD being the leading cause of mortality and morbidity in Saudi Arabia [36], it is important to examine preventive healthcare services to address this burden.

### Predisposing factors

#### Sex.

Sex emerged as the most common significant predisposing factor. Female participants demonstrated higher tendencies to receive PreDM and T2DM screening, weight-loss counseling, healthy-diet and physical activity counseling, and statin therapy. However, they were less likely to receive smoking-cessation counseling.

Despite a marginally higher proportion of female over male respondents, evidence suggests that female individuals tend to utilize healthcare services more frequently [37, 38]. Female individuals generally exhibit higher awareness of health and are more engaged in preventive behaviors [39]. However, despite their proactive health habits, female individuals tend to experience worse outcomes and higher mortality rates following cardiovascular events compared to male individuals, who have a higher incidence of CVD [40, 41].

In the context of the Kingdom of Saudi Arabia, several sociocultural dynamics may still influence healthcare utilization, such as reliance on male guardians and mobility constraints [42]. Our findings indicate a strong positive association between sex and the utilization of many PCCS. However, female individuals were less likely to receive smoking-cessation counseling. Despite the relatively low prevalence of smoking among women (4.2% as of 2019) [43], societal stigma and HCPs’ reluctance to screen for smoking in female individuals exacerbate the challenge. These challenges often stem from stigmatization of female smokers and hesitancy to disclose smoking, further exacerbated by tobacco marketing strategies that normalize smoking across different communities [44].

#### Parenthood.

Having children significantly influenced the utilization of PCCS in several models, including PreDM and T2DM screening, weight-loss counseling, and statin use. The effect of parenthood can be attributed to a combination of behavioral and social factors.

First, the incidence of obesity has been reported to increase by ~4% with each additional child [45], suggesting a direct impact of parenthood on physical health conditions that might necessitate more healthcare utilization. Conversely, parental responsibility often increases personal health awareness, promoting greater engagement with healthcare services [46]. Moreover, parents might adopt a healthy lifestyle mainly to serve as role models for their children. One study emphasized how parental influence can significantly impact children’s health behaviors through role modeling [47]. Additionally, parents’ active involvement in community and social networks facilitates greater health consciousness, as the prevalence of chronic diseases within these networks often encourages the adoption of healthy behaviors. Altogether, these factors demonstrate how parenthood serves as an incentive for increased healthcare utilization.

#### Education and income.

Education served as a significant variable in three models; individuals with lower education levels were more likely to receive waist-circumference screening and weight-loss counseling, while those with higher education levels were more often counseled on physical activity. The analysis revealed that individuals with lower educational attainment are more likely to engage in obesity-related services. This trend aligns with broader research findings that demonstrate a correlation between lower educational levels and higher obesity rates, necessitating greater service utilization. A study aggregating data from 1985 to 2015 identified a distinct association between lower educational levels and an elevated lifetime risk of CVD, with a hazard ratio of 1.58 (95% CI, 1.38–1.80) for those with less than high school education [48].

In the context of healthcare, educational attainment is intricately linked to socioeconomic factors such as income and employment status. Despite the nonsignificant impact of employment status across all models, lower income was found to be associated with healthy-diet counseling, highlighting the direct link between socioeconomic status (SES) and physical health. Research has found that individuals with lower SES have a higher incidence of CVD [49], and are more likely to utilize primary healthcare services, while those with higher SES tend to seek more specialized healthcare services [50, 51].

In Saudi Arabia, where healthcare services are provided free of charge for Saudi nationals, income has been an insignificant factor in healthcare utilization in multiple other studies [52, 53]. The complexities of SES, including the challenges it poses in implementing lifestyle changes [54], remain critical areas for policy intervention to enhance healthcare outcomes and utilization.

#### Age.

In our hypertension model, age ≥ 35 years emerged as a significant factor. Generally, blood pressure-screening, while a routine procedure at AFHS facilities, is particularly emphasized for older adults due to the increased prevalence of chronic conditions [13]. The prevalence of hypertension in Saudi Arabia exhibits a distinct age-related trend [55], which reflects broader global trends but is particularly pronounced locally due to demographic shifts and lifestyle factors [56].

In 2019, individuals aged 65 and above comprised 2.4% of Saudi Arabia’s total population; however, current projections estimate this demographic will reach 21% by 2050 [57]. In response to these major demographic changes, several national health initiatives have evolved to cater to an aging population. The Saudi Ministry of Health has implemented five comprehensive programs aimed at addressing chronic conditions that predominantly affect older adults [58]. Such initiatives are fundamental to the current Saudi Model of Care under the National Health Transformation Program [7], showcasing a strategic turn towards enhancing healthcare services to mitigate age-related conditions.

### Enabling factors

Among the seven enabling factors analyzed, six had a significant impact. Despite Saudi Arabia’s healthcare system, which follows a universal coverage scheme, access to services provided by AFHS remains exclusive to military personnel, their dependents, and workers under the Ministry of Defense.

Notably, the current Saudi healthcare system allows for the possibility of redundant service provision, where two public service providers could offer identical services simultaneously to the same individual. Here, we present the enabling variables encountered during the patient journey after confirming that all services were offered at AFHS PHCs exclusively.

#### Social support.

Social support emerged as a significant factor in the hypertension screening model. Perceived social support, defined as the subjective evaluation of the availability of material and psychological support from one’s social environment —including family and friends [59]— has consistently been used as a predictor of self-reported disease outcomes, including hypertension [60]. Interestingly, individuals who perceive high levels of social support are more likely to engage in preventive health services; this includes those who have a spouse or children [61].

In Saudi Arabia, social support is a deeply ingrained cultural value. This support plays a vital role in health management as it extends beyond merely facilitating transportation to healthcare facilities. It includes aiding in navigating complex health systems, ensuring medication adherence, and providing reminders for appointments. Moreover, social support can influence health behaviors as encouragement and motivation from family and friends often lead to positive health behavior changes [62], all of which can affect healthcare utilization.

#### Easy appointment booking.

The ease of booking appointments at AFHS was identified as a significant enabling factor in the weight-loss counseling model, increasing the likelihood of accessing PCCS through improved patient facilitation, which is directly related to service utilization.

Globally, the shift towards electronic medical appointment scheduling reflects a broader trend aimed at enhancing patient-centeredness by simplifying access to care and reducing wait times, thereby improving overall healthcare outcomes and patient retention [63]. However, booking preferences among different service users can vary and may change over time [64], which occasionally presents as barriers such as the unavailability of suitable time slots. These limitations can deter new patients from utilizing PCCS, thereby negatively affecting the uptake of essential preventive services [65]. Another factor is related to the technological illiteracy among older adults, which may hinder their ability to book appointments by themselves, although telephone scheduling options remain available at AFHS.

#### Vehicle ownership.

Vehicle ownership emerged as a significant factor across three distinct analytical models. Specifically, Model-3 and Model-4 showed positive associations, with vehicle ownership correlating with increased likelihood of waist-circumference screening and statin use. Conversely, Model-6 revealed a negative correlation, indicating that vehicle ownership may decrease the likelihood of engaging in physical activity counseling.

Universally, the availability of personal transportation has been identified as a critical enabler for accessing healthcare services. Studies have consistently found that the lack of reliable transportation correlates with lower medication adherence and reduced attendance at medical appointments [66]. These findings are particularly relevant in Riyadh, where residents heavily rely on personal vehicles due to the expansive urban layout of the city and the limited public transportation options. This enabler is even more crucial for populations facing mobility restrictions, such as older adults who may have more disabilities, illnesses, and a greater need for frequent healthcare visits [67]. Notably, in 2020, female drivers in Saudi Arabia constituted one-third of total drivers according to 2018 projections [68]. Despite the royal resolution in 2018, which allowed women to drive, female non-drivers may still face barriers in accessing healthcare due to their dependency on available private or public transport.

#### Distance to PHC.

Individuals who traveled for more than one hour were more likely to utilize hypertension screening and waist-circumference services. Globally, our results align with findings from a 2013 study in China, where residents living far from hospitals were more likely to utilize health services, often with greater frequency [69]. This correlation confirms the global relevance of geographic barriers to healthcare access. However, our study’s respondents lived solely within Riyadh, which does not allow for a direct comparison between urban and rural healthcare utilization patterns. Nevertheless, studies in OECD member countries have demonstrated that individuals in remote rural areas often face significant healthcare challenges related to chronic diseases, further emphasizing the importance of addressing travel time as a critical enabling factor in access [70]. Fortunately, the AFHS has an extensive network of PHCs spread around Riyadh; however, this network does not extend into the rural areas surrounding the city. This insight is relevant for AFHS strategies, as geographical dispersion may influence engagement and frequency of access.

#### Language barriers.

Language barriers can negatively impact individuals’ satisfaction with care and communication with HCPs [71]. In the final hypertension-screening model, language barriers were associated with higher screening utilization. Moreover, individuals facing language barriers often experience poor health outcomes [72]. In the Kingdom, this can lead to misunderstanding, misdiagnosis, and medication errors [73].

This adverse impact on health outcomes may lead to increased utilization of healthcare services, which in turn adds to financial strain on the system [74]. On the other hand, patients experiencing language barriers frequently report lower satisfaction levels, which might deter them from seeking healthcare services [71], including PCCS. Addressing these barriers through training and implementing language-support services can enhance patient satisfaction, improve utilization, and lead to better health outcomes [75].

#### Waiting time.

Pre-consultation waiting time influenced the utilization of PreDM and T2DM screening services. Individuals waiting <30 minutes utilized blood glucose screening more frequently. Global research findings show that shorter waits increase satisfaction and can improve utilization [76]. Individuals may even prefer private over public healthcare centers for their shorter waiting times [77]. Effective waiting time management has been shown to improve not only patient satisfaction but also overall clinical outcomes and service efficiency [78]. This can be achieved by implementing more advanced appointment scheduling systems and optimizing staffing and patient-flow management techniques [79].

### Need factors

In nearly all the services studied for utilization, a family history of CVD, individual CVD risk factors, and perceived health status were identified as significant need factors.

#### Family history of CVD.

Familial predisposition, specifically having a first-degree relative with CVD, was associated with a higher likelihood of hypertension screening. Notably, 90% of our respondents reported a positive family history of CVD. Although this factor was significant only in the hypertension screening model, familial predisposition is recognized as a risk factor for multiple chronic conditions. Family history plays a crucial role in CVD risk assessment and is incorporated into many risk calculators [80]. However, many individuals report that their familial CVD history was not addressed in consultations [81], which might affect their understanding of service need. Notably, a study found that awareness of family history of CVD or personal risk for CVD alone does not reliably predict changes in health-related behaviors [82].

#### CVD risk factors.

Individuals with CVD risk factors were more likely to use PreDM and T2DM screening, waist-circumference screening, and weight-loss counseling according to our models. This significant association might suggest an increased level of awareness among individuals about their elevated risk of CVD due to perceived vulnerability. Such awareness is likely to be amplified by HCPs who stress the importance of regular monitoring, thereby triggering more intensive healthcare interactions. HCPs might prioritize these patients for frequent reviews and screenings due to the potential severity of their conditions [83]. Given the high prevalence of CVD in the Saudi population, the AFHS provides its beneficiaries with a higher level of care through Chronic Illness Clinics. These clinics manage multimorbidity more closely, with a holistic team that includes diabetic educators, clinical pharmacists, and dietitians at WHC. This integrated care approach is essential not only for optimal disease management but also for prevention of secondary conditions, and may increase rates of screening and counseling.

#### Perceived health status.

Perceived health status is distinct from evaluated health status. Research has shown that individuals who perceive their health as poor are more likely to utilize healthcare services [84]. This was evident in our results, where perceived health status was a significant factor in weight-loss counseling and smoking-cessation counseling. Although this measure is subjective, it is considered a reliable predictor of mortality [85]. Several material, psychosocial, and behavioral factors play an integral role in self-reported health status [86]. Notably, those who perceive their health status as less than excellent were more likely to receive treatment than those with excellent self-rated health status [87]. Perceived health status could also affect patient satisfaction with healthcare services [88]. All these factors work concurrently to explain the utilization of PCCS in this community.

### Limitations

Survey-based studies are well known for several limitations. Self-reported data may be subject to recall and/or response bias. Respondents may not have accurately recalled whether they had received the service within the timeframe covered by the questionnaire. Additionally, the BMHSU is a multilevel framework that encompasses a wide range of factors, including contextual ones, which adds to the complexity of the model. Although we examined 18 different factors, associations might have been influenced by other indirect effects. As noted, contextual factors were not taken into consideration during our analysis. Thirdly, preventive cardiovascular services were limited to those recommended in two national and international guidelines. The PHA Preventive Clinical Guidelines share many similarities with the USPSTF guidelines; however, some physicians may rely on other references. Both guidelines utilize Atherosclerotic Cardiovascular Disease (ASCVD) risk scores, which incorporate lipid panel, aspirin use, and blood-pressure measurements, factors that were not included in our survey instrument. Finally, the USPSTF guidelines clearly state that their recommendations apply to asymptomatic individuals; however, 53% of our sample reported having CVD. Additionally, because a convenience sampling technique was used, this approach may have limited the representativeness of the sample, thereby affecting the generalizability of the findings to the broader population. Furthermore, due to the nature of cross-sectional studies, causal relationships cannot be established.

## Conclusion and recommendations

Grounded in Andersen’s model of healthcare utilization, our study examined 18 determinants and demonstrated complex, interconnected associations among several of them. Our models identified factors warranting special consideration. Predisposing factors, although the most prevalent and least modifiable, necessitate strategies tailored to distinct population segments. By contrast, enabling factors should inform organizational planning and financial frameworks within PHCs. Need factors reflect individual-level clinical and experiential constraints that HCPs can proactively address.

Within Saudi Arabia, beneficiaries of AFHS represent a unique demographic group. The findings of our study support previous research and will contribute to a more granular understanding of how to optimize PCCS; however, further objective research is needed. Future efforts should integrate machine learning techniques to deepen the segmentation of the AFHS population based on predisposing factors, which would allow for the development of tailored programs that specifically address the unique needs of the AFHS population. Integrating patient journey mapping within AFHS PHCs would identify critical access barriers. Employing longitudinal studies allows for observing changes in utilization patterns over time, accompanied by routine assessment and stakeholders’ feedback. Collectively, these insights should inform institutional and national health policies aimed at strengthening preventive care delivery and optimizing the utilization of preventive services by ensuring that social barriers are taken into consideration.

## Supporting information

S1 Appendix Data collection questionnaire (English and Arabic versions). This appendix includes the original study questionnaire in both English and Arabic languages used for data collection.
(PDF)
