# Supplementary material for: Examining the role of social factors in the utilization of preventive cardiovascular clinical services: An analysis using Andersen’s behavioral health model
Source: PLoS One. 2026 Jan 23;21(1):e0341074. doi: 10.1371/journal.pone.0341074 (PMC12829952; doi:10.1371/journal.pone.0341074)
Supplement: S1 Appendix — This appendix includes the original study questionnaire in both English and Arabic languages used for data collection. (PDF) [file pone.0341074.s001.pdf]

Studying the Utilization of Preventive Cardiovascular Services
At Ministry of Defense's Primary Healthcare Centers

**Dear visitor,**<br>I extend my deepest thanks and appreciation for your generous contribution and your valuable time to participate in this project. <u>This survey takes less than 5 minutes to complete.</u>	
1	**Study objectives and benefits:**<br>- This study aims to identify the factors that can assist or hinder the use of preventive cardiovascular clinical services (e.g., blood pressure, blood sugar, and lipids screenings) in order to facilitate the provision and use of such services.
2	**Voluntary participation:**<br>- Your participation in this study is voluntary, you have the right not to participate or to withdraw at any time for any reason, and your answers will not be used. There are no unexpected risks to your health if you agree to participate.
3	**Confidentiality of collected data:**<br>- <u>This survey does not collect any personal information</u> and you can not be identified through your answers. The responses provided in this survey will be retained and will not be viewed by people outside the research team. The results will be analyzed and the study will be published in a scientific journal later.<br>- This study has been approved by the Research Ethics Committee at the Scientific Research Center (SRC) at Prince Sultan Military Medical City with number: (E-2274)
4	**If you have any questions, please do not hesitate to contact the principal investigator:**<br>- Dr. Sultan Alamri. Preventive Medicine Program Resident at Prince Sultan Military Medical City.<br>- Email: [redacted]
**By proceeding to answer, you confirm that you have read and agreed to participate.**	

— BEGINNING OF SURVEY —

1	Did you have an appointment at the **General Family Medicine Clinic** in this center **<u>TODAY</u>**?	○ Yes	○ No
2	Have you **<u>EVER</u>** visited a **General Family Medicine Clinic** at this center?	○ Yes	○ No

3	Please select your **sex**:	○ Male	○ Female
4	**<u>FOR WOMEN:</u>** Are you currently pregnant?	○ Yes	○ No

5	Do you **currently** have any **cardiovascular disease?**	○ Yes	○ No

6	Please **write your age**:	

7	Please write your **weight in kilograms:**	
8	Please write your **height in centimeters:**	

Part 1: Socio-demographic factors:

1	Please specify your **nationality**:	◯ Saudi	◯ Non-Saudi (Expatriate)

2	What is the **highest** level of education you received?	◯ No formal education
		◯ Middle school degree or less
		◯ High-school degree
		◯ Bachelor's degree
		◯ Higher than Bachelor's degree

3	What is your **current** marital status?	◯ Married	◯ Single (never married)
		◯ Divorced	◯ Widowed

4	How many **children** do you have?	◯ None	◯ 1-2 Children
		◯ 3-4 Children	◯ More than 4 children

5	Where is your **current** place of residence?	◯ **Inside** city of Riyadh	◯ **Outside** city of Riyadh

6	**IF YOU CHOSE 'Outside city of Riyadh':** Please write your place of residence:	

7	Please select your **current employment status**:	◯ Employed (governmental/private sectors)
		◯ Retired
		◯ Unemployed (Not a student)
		◯ Student
		◯ Self-employed

8	What is your average **household** income?	◯ **Less than** 5000 Saudi Riyals
		◯ From 5000 **to** 10,000 Saudi Riyals
		◯ From 10,000 **to** 15,000 Saudi Riyals
		◯ **More than** 15,000 Saudi Riyals

Part 2: Use of Preventive Cardiovascular Clinical Services:

1	**WITHIN THE PAST 5 YEARS:**<br>Have you ever had your **blood pressure checked**<br>at **any of** Ministry of Defense's Primary Healthcare Centers?	◯ Yes	◯ No

2	**WITHIN THE PAST 5 YEARS:**<br>Have you ever been done a **blood lipid measurements** (Cholesterol)<br>at **any of** Ministry of Defense's Primary Healthcare Centers?	◯ Yes	◯ No
3	Have you ever been **offered anti-lipids medication**<br>at **any of** Ministry of Defense's Primary Healthcare Centers?	◯ Yes	◯ No

4	**WITHIN THE PAST 3 YEARS:**<br>Have you ever had your **blood sugar checked** (either fasting blood glucose, HbA1c, or glucose tolerance test)<br>at **any of** Ministry of Defense's Primary Healthcare Centers?	◯ Yes	◯ No

5	Have you ever been **counselled about obesity**<br>at **any of** Ministry of Defense's Primary Healthcare Centers?	◯ Yes	◯ No
6	**WITHIN THE PAST 2 YEARS:**<br>Have your ever been **offered to take your waist circumference measurements** to check abdominal obesity<br>at **any of** Ministry of Defense's Primary Healthcare Centers?	◯ Yes	◯ No

7	Have you even been **counselled about healthy diet**<br>at **any of** Ministry of Defense's Primary Healthcare Centers?	◯ Yes	◯ No
8	Have you even been **counselled about physical activity**<br>at **any of** Ministry of Defense's Primary Healthcare Centers?	◯ Yes	◯ No

9	Are you a **currently a smoker** or are you **regularly exposed to people who smoke?**	◯ Yes	◯ No
10	**IF YOU WERE A SMOKER OR REGULARLY EXPOSED TO SMOKING:**<br>Have you even been **counselled** about **smoking cessation**<br>at **any of** Ministry of Defense's Primary Healthcare Centers?	◯ Yes	◯ No

11	**IF YOU ARE A SMOKER ABOVE 65 YEARS OLD:**<br>Have you even been **offered Ultrasounds** for the risk of **Abdominal Aortic Aneurysm**<br>at **any of** Ministry of Defense's primary healthcare centers?	◯ Yes	◯ No

Part 3: Enabling and need factors to use Preventive Cardiovascular Clinical Services:

1	Do you have a **supplementary private health insurance**?	◯ Yes	◯ No

2	**MOST OF THE TIMES:**<br>Do you have **someone** who **can help you** when you are **sick?**	◯ Yes	◯ No

3	**MOST OF THE TIMES:**<br>Do you find it **easy to schedule an appointment** at Ministry of Defense's Primary Healthcare Centers?	◯ Yes	◯ No

4	Do you have a **regular access to a vehicle for transportation** for your appointments at Ministry of Defense's Primary Healthcare Centers?	◯ Yes	◯ No
5	**ON AVERAGE:**<br>**How close** is the **nearest** Ministry of Defense Primary Healthcare Centers **to your current residence?**	◯ **Less than** 30 minutes<br>◯ 30 minutes **to** 1 hour<br>◯ 1 **to** 2 hours<br>◯ **More than** 2 hours	

6	**ON AVERAGE:**<br>**How long** do you **typically wait in the waiting room** after you arrive at **any of** Ministry of Defense's Primary Healthcare Centers **before seeing the doctor?**	◯ **Less than** 15 minutes<br>◯ 15 **to** 30 minutes<br>◯ 31 minutes **to** 1 hour<br>◯ **More than** 1 hour

7	Have you ever experienced **language barriers** when communicating with **healthcare staff** at **any of** Ministry of Defense's Primary Healthcare Centers?	◯ Yes	◯ No
8	**ON AVERAGE:**<br>Do you feel that you **received high-quality medical care** during your visit at **any of** Ministry of Defense's Primary Healthcare Centers?	◯ Yes	◯ No

9	Do you have a **first-degree family history of cardiac diseases**? (e.g., parents, siblings, or offspring)	◯ Yes	◯ No
10	Have you ever been **diagnosed** with any of these following conditions during any point of your life:<br>- High blood pressure - High blood cholesterol<br>- Diabetes	◯ Yes	◯ No

11	**How would you** generally **rate your health**?	◯ **Excellent** health	
		◯ **Very good** health	◯ **Good** health
		◯ **Fair** health	◯ **Poor** health

— END OF SURVEY, THANK YOU —

أو يمكنك استخدام الهاتف للإجابة

دراسة عوامل **استخدام الخدمات الوقائية لأمراض القلب**
في عيادات طب الأسرة التابعة لوزارة الدفاع

**عزيزي الزائر:**<br>أتقدم لسعادتكم بوافر الشكر والتقدير على منحكم جزءًا من وقتكم الثمين للمشاركة في هذا الاستبيان. يستغرق هذا الاستبيان أقل من ٥ دقائق للإجابة عليه كاملًا.

1	**أهداف الدراسة وفوائدها:**<br>- تهدف هذه الدراسة إلى تحديد العوامل التي تحفز أو تثبط من استخدام الخدمات الطبية الوقائية لأمراض القلب (مثل فحص ضغط الدم والسكر والدهون في الدم) من أجل تقديم واستخدام تلك الخدمات من قبل الزوار.
2	**طوعية المشاركة:**<br>- مشاركتك في هذه الدراسة اختيارية، ولك كامل الحق في عدم المشاركة أو الانسحاب في أي وقت تشاء لأي سبب من الأسباب، ولن يتم استخدام إجاباتك. لا توجد مخاطر غير متوقعة على سلامتك اذا وافقت على المشاركة.
3	**سرية البيانات:**<br>- هذا الاستبيان لا يقوم بجمع أي معلومات شخصية ولن يمكن التعرف عليك من خلال أجوبتك، سيتم الاحتفاظ بالإجابات المقدمة في هذا الاستبيان ولن يتم الإطلاع عليها من أشخاص خارج فريق البحث، سيتم تحليل النتائج ونشر الدراسة في مجلة علمية لاحقًا.<br>- تمت الموافقة على هذه الدراسة من لجنة أخلاقيات البحث في مركز الأبحاث العلمية بمدينة الأمير سلطان الطبية العسكرية برقم: (E-2274)
4	**إذا كانت لديك أي أسئلة، لا تتردّد بالتواصل مع الباحث الرئيسي:**<br>- د. سلطان العمري، طبيب مقيم ببرنامج الطب الوقائي في مدينة الأمير سلطان الطبية العسكرية.<br>- البريد الالكتروني: [redacted]

بمتابعتك في الإجابة على هذا الاستبيان فأنت تقر بإطلاعك على ما سبق وتوافق على المشاركة.

— بداية الاستبيان —

1	هل كانت زيارتك **اليوم** **في عيادات طب الأسرة العامة؟**	◯ نعم	◯ لا
2	**هل سبق أن** زرت عيادة طب الأسرة **العامة** في هذا المركز الصحي؟	◯ نعم	◯ لا

3	يرجى تحديد **الجنس:**	◯ ذكر	◯ أنثى
4	**للسيدات**: هل أنتِ حامل حاليًا؟	◯ نعم	◯ لا

5	هل تعاني **حاليًا** من أي **أمراض في القلب** أو **الأوعية الدموية؟**	◯ نعم	◯ لا

6	**يرجى كتابة عمرك:**	

7	ما هو **وزنك** بالكيلوجرام؟	
8	ما هو **طولك** بالسنتيمتر؟	

الجزء الأول: العوامل الاجتماعية والديموغرافية:

1	يرجى تحديد **جنسيتك**:	◯ سعودي	◯ غير سعودي (مقيـم)

2	ما هو **أعلى مستوى تعليمي** حصلت عليه؟	◯ لم أتلقَ أيّ تعليم رسمي
		◯ تعليم المرحلة المتوسطة أو أقل
		◯ شهادة المرحلة الثانوية
		◯ درجة البكالوريوس
		◯ درجة أعلى من درجة البكالوريوس

3	ما هي حالتك الاجتماعية **الحالية**؟	◯ متزوج أو متزوجة	◯ أعزب أو عزباء (لم أتزوج أبدًا)
		◯ مطلق أو مطلقة	◯ أرمل أو أرملة

4	كم عدد **أطفالك**؟	◯ لا يوجد	◯ ١-٢ أطفال
		◯ ٣-٤ أطفال	◯ أكثر من ٤ أطفال

5	ما هو مكان إقامتك **حاليًا**؟	◯ **داخل** مدينة الرياض	◯ **خارج** مدينة الرياض

6	**إذا كنت تسكن خارج مدينة الرياض**<br>الرجاء كتابة المنطقة:	

7	ما هو وضعك الوظيفي **الحالي**؟	◯ موظف في قطاع حكومي أو خاص
		◯ متقاعد
		◯ عاطل عن العمل ولست طالبًا
		◯ طالب
		◯ أعمل لحسابي الخاص (مهنة حرة)

8	ما هو **مجموع دخل أسرتك**؟	◯ **أقل من** 5,000 ريال سعودي
		◯ من 5,000 **إلى** 10,000 ريال سعودي
		◯ من 10,000 **إلى** 15,000 ريال سعودي
		◯ **أكثر من** 15,000 ريال سعودي

الجزء الثاني: استخدام الخدمات الطبية الوقائية لأمراض القلب:

1	**خلال الخمس سنوات الماضية:**<br>هل سبق أن قمت **بقياس ضغط الدم**<br>**في أي مركز** من مراكز طب الأسرة التابعة لوزارة الدفاع؟	◯ نعم	◯ لا

2	**خلال الخمس سنوات الماضية:**<br>هل سبق أن قمت **بتحليل نسبة الدهون** في الدم (الكولسترول)<br>**في أي مركز** من مراكز طب الأسرة التابعة لوزارة الدفاع؟	◯ نعم	◯ لا
3	هل سبق إخبارك بأنك تحتاج **أدوية لعلاج ارتفاع الدهون في الدم**<br>**في أي مركز** من مراكز طب الأسرة التابعة لوزارة الدفاع؟	◯ نعم	◯ لا

4	**خلال الثلاث سنوات الماضية:**<br>هل سبق أن قمت بفحص **نسبة السكر في الدم** (سواءً السكر التراكمي أو الصائم أو اختبار تحمل السكر)<br>**في أي مركز** من مراكز طب الأسرة التابعة لوزارة الدفاع؟	◯ نعم	◯ لا

5	هل سبق أن تلقيت **استشارة بخصوص السمنة**<br>**في أي مركز** من مراكز طب الأسرة التابعة لوزارة الدفاع؟	◯ نعم	◯ لا
6	**خلال العامين الماضيين:**<br>هل سبق أن عُرض عليك إجراء **قياس لمحيط الخصر** لقياس سمنة البطن<br>**في أي مركز** من مراكز طب الأسرة التابعة لوزارة الدفاع؟	◯ نعم	◯ لا

7	هل سبق أن تلقيت مشورة بشأن **اتباع نظام غذائي صحي**<br>**في أي مركز** من مراكز طب الأسرة التابعة لوزارة الدفاع؟	◯ نعم	◯ لا
8	هل سبق أن تلقيت مشورة بشأن **ممارسة النشاط البدني**<br>**في أي مركز** من مراكز طب الأسرة التابعة لوزارة الدفاع؟	◯ نعم	◯ لا

9	هل أنت **مدخن** أو **تخالط أشخاص مدخنين بانتظام؟**	◯ نعم	◯ لا
10	**إذا كنت مدخناً أو تخالط أشخاصًا مدخنين بانتظام:**<br>هل نصحك أي ممارس صحي **بشأن الإقلاع عن التدخين أو مخالطة المدخنين**<br>**في أي مركز** من مراكز طب الأسرة التابعة لوزارة الدفاع؟	◯ نعم	◯ لا

11	**إذا كنت مدخناً وعمرك يتجاوز 65 عامًا:**<br>هل عرض عليك إجراء **أشعة الموجات فوق الصوتية** لتحديد مدى خطر الإصابة **بتمدد الشريان الأورطي البطني** (Abdominal Aortic Aneurysm)<br>**في أي مركز** من مراكز طب الأسرة التابعة لوزارة الدفاع؟	◯ نعم	◯ لا

الجزء الثالث: عوامل التمكين والاستفادة من الخدمات الطبية الوقائية لأمراض القلب:

1	هل يوجد لديك **تأمين صحي خاص إضافي**؟	◯ نعم	◯ لا

2	**في أغلب الأوقات:**<br>هل لديك **شخص يمكنه رعايتك** عندما تكون **مريضًا**؟	◯ نعم	◯ لا

3	**في أغلب الأوقات:**<br>هل تجد أنه **من السهل حجز موعد** في مراكز طب الأسرة التابعة لوزارة الدفاع؟	◯ نعم	◯ لا
4	**في أغلب الأوقات:**<br>هل تتوفر لديك **سيارة للذهاب إلى مواعيدك** في مراكز طب الأسرة التابعة لوزارة الدفاع؟	◯ نعم	◯ لا

5	**في أغلب الأوقات:**<br>ما هو **متوسط المسافة بين** مكان إقامتك الحالي **وأقرب مركز طب أسرة** تابع لوزارة الدفاع؟	◯ **أقل** من 30 دقيقة	◯ من 30 دقيقة **إلى** ساعة
		◯ من ساعة **إلى** ساعتين	◯ **أكثر** من ساعتين

6	**في أغلب الأوقات:**<br>ما هو **متوسط المدة التي تنتظرها في غرفة الانتظار قبل الدخول على طبيب الأسرة في أي مركز** من مراكز طب الأسرة التابعة لوزارة الدفاع؟	◯ **أقل من** 15 دقيقة	◯ من 15 **إلى** 30 دقيقة
		◯ من 31 دقيقة **إلى** ساعة	◯ **أكثر** من ساعة

7	هل سبق أن واجهت **صعوبات لغوية** عند التواصل **مع الممارسين الصحيين في أي مركز** من مراكز طب الأسرة التابعة لوزارة الدفاع؟	◯ نعم	◯ لا
8	**في أغلب الأوقات:**<br>هل تشعر أنك **تلقيت رعاية طبية عالية الجودة** خلال زيارتك **لأي مركز من مراكز طب الأسرة** التابعة لوزارة الدفاع؟	◯ نعم	◯ لا

9	هل لديك **أفراد عائلة** مصابون **بأمراض القلب** من **الدرجة الأولى** (آباء أو أشقاء أو أبناء)؟	◯ نعم	◯ لا

10	**هل تم تشخيصك في أي وقت من عمرك** بأي من الحالات الطبية التالية:<br>• ارتفاع ضغط الدم<br>• ارتفاع نسبة الدهون (الكوليسترول) في الدم<br>• داء السكري	◯ نعم	◯ لا

11	ما هو **تقييمك لحالتك الصحية بشكل عام**؟	◯ حالتي الصحية **ممتازة**
		◯ حالتي الصحية **جيدة جدًا**
		◯ حالتي الصحية **جيدة**
		◯ حالتي الصحية **لا بأس بها**
		◯ حالتي الصحية **سيئة**

- انتهى الاستبيان، شكرًا لكم. -
